# Intra-Articular Monoclonal Antibodies in Temporomandibular Joint Disorders: Current Evidence

**DOI:** 10.3390/cells15151410

**Published:** 2026-08-04

**Authors:** Zuzanna Baniak, Maciej Chęciński, Wojciech Macek, Maja Kosińska, Amelia Hoppe, Julia Kasprzycka, Oliwia Jagiełło, Izabella Chyży, Karolina Grzybowska-Kowalczyk, Tomasz Horodniczy, Kamila Chęcińska, Maciej Sikora

**Affiliations:** 1 Stomatologia Centrum, Moniuszki 3/7, 32-020 Wieliczka, Poland; baniakzuzanna@gmail.com; 2 National Medical Institute of the Ministry of the Interior and Administration, Wołoska 137, 02-507 Warsaw, Poland; maciej.checinski@pimmswia.gov.pl (M.C.); ichyzy@gcm.pl (I.C.); maciej.sikora@pimmswia.gov.pl (M.S.); 3 Department of Maxillofacial Surgery, Hospital of the Ministry of the Interior and Administration, Wojska Polskiego 51, 25-375 Kielce, Poland; 4 Department of Oral Surgery, Preventive Medicine Center, Komorowskiego 12, 30-106 Cracow, Poland; ld.wojciech.macek@wp.pl (W.M.); majaakosinska@gmail.com (M.K.); amelia.a.hoppe@gmail.com (A.H.); 5 Miodowa Clinic, Głowaccy Medical and Dental Practice Professional Partnership, Miodowa 2, 62-090 Rokietnica, Poland; lekdentkasprzycka@gmail.com; 6 Gabinety Lekarskie Dent-im M.B. Pawelczyk Spółka Jawna, Różana 13/1, 61-577 Poznań, Poland; oliwiajagiellodent@gmail.com; 7 Uśmiech Family Dental Clinic, Nastrojowa 26, 91-496 Łódź, Poland; karolina.orto.gk@gmail.com; 8 Ortho.pl Dental Office, Buforowa 34, 52-131 Wrocław, Poland; tomasz.horodniczy2@gmail.com; 9 Department of Biochemistry and Medical Chemistry, Pomeranian Medical University, Powstańców Wielkopolskich 72, 70-111 Szczecin, Poland

**Keywords:** temporomandibular disorders, temporomandibular joint, monoclonal antibodies, infliximab, intra-articular administration, TNF-α inhibition, biologic therapy, inflammatory arthropathy

## Abstract

**Highlights:**

**What are the main findings?**

- Only four heterogeneous studies met the eligibility criteria, and all investigated local TNF-α blockade; no eligible intra-articular TMJ studies examined NGF, CGRP, or interleukin targets.
- Clinical symptomatic improvement was limited to a single adult case, while pediatric MRI abnormalities predominantly persisted or worsened; supportive anti-inflammatory findings were confined to a rabbit model.

**What are the implications of the main findings?**

- The available evidence is hypothesis-generating rather than practice-changing and does not support the routine clinical use of intra-articular monoclonal antibodies in TMJ disorders.
- Further translation should prioritize TMJ-specific formulation, pharmacokinetic, and safety studies before clinical trials in highly selected patients with refractory immune-mediated inflammatory TMJ arthritis.

**Abstract:**

Immune-mediated temporomandibular joint disorders involve cytokine-driven inflammation and may represent a target for biologic therapy. This paper summarizes the evidence on intra-articular monoclonal antibody administration in temporomandibular joint disorders. PubMed, Europe PMC, and BASE were searched from inception to 7 April 2026 for clinical and preclinical studies evaluating intra-articular monoclonal antibodies in the temporomandibular joint. Four studies were identified: two retrospective clinical studies, one case report, and one preclinical study. All four studies investigated local TNF-α blockade: the three clinical studies used infliximab, whereas the preclinical study used an anti-rabbit TNF-α monoclonal antibody. Symptomatic benefit was confined to one adult case and anti-inflammatory effects to the preclinical study, whereas pediatric MRI abnormalities persisted or worsened, providing no evidence of structural efficacy. The evidence remains limited, heterogeneous, and exploratory. Intra-articular monoclonal antibody therapy is biologically plausible, but its clinical efficacy and safety require further investigation.

## 1. Introduction

Targeted biologic therapy has emerged as a potential direction in the management of inflammatory temporomandibular joint disorders, although the supporting evidence remains sparse and heterogeneous. Temporomandibular disorders (TMDs) commonly cause pain and functional impairment [1]. Inflammation contributes to their pathophysiology through cytokine-mediated synovitis, cartilage degradation, and nociceptive signaling [2]. TMJ involvement is particularly relevant in rheumatoid and psoriatic arthritis [3,4,5] and juvenile idiopathic arthritis [6,7]. However, current therapies remain largely symptomatic [8,9].

Increasing attention has therefore focused on molecular targets involved in inflammation, pain, and joint remodeling [8,9], including TNF-α, IL-1β, and IL-6 signaling [2,10,11]. Monoclonal antibodies can selectively inhibit such pathways [12]. TNF-α inhibitors have shown potential in inflammatory joint disease and TMJ-related symptoms [3], with supporting evidence from other joints [12,13], although immunogenicity and adverse effects remain concerns [14,15]. Although nerve growth factor (NGF) and calcitonin gene-related peptide (CGRP) are mechanistically relevant to TMD-related pain [16,17], neither has been evaluated using intra-articular monoclonal antibodies in the TMJ, and both are discussed as future directions rather than current evidence.

Local intra-articular administration could provide targeted modulation within the TMJ [12]. Preliminary reports describe intra-articular infliximab in psoriatic arthritis [18] and refractory pediatric TMJ arthritis [6], but imaging outcomes remain inconsistent [7]. Against this background, recent syntheses of autologous conditioned serum and other standalone intra-articular interventions highlight an expanding but still incompletely defined therapeutic landscape, supporting further evaluation of targeted biologic approaches in TMDs [19,20].

This paper summarizes the available evidence, evaluates its biological and translational rationale, and identifies priorities for further research.

## 2. Materials and Methods

The protocol was prospectively registered in the Open Science Framework (osf.io/tdfra). PubMed, Europe PMC, and BASE were searched from inception to 7 April 2026 for clinical and preclinical studies on intra-articular monoclonal antibody administration in the temporomandibular joint. The selection of information sources was intentionally restricted to freely accessible platforms to minimize access-related barriers to independent replication. This combination provided biomedical coverage through PubMed and Europe PMC and broader multidisciplinary and repository-based coverage through BASE. Records were independently screened, and data on study design, population or experimental model, monoclonal antibody, dose and administration, outcomes, and safety were independently charted; methodological quality was then assessed. Detailed eligibility criteria, search strategy, study selection, data charting, appraisal procedures, and synthesis methods are provided in the Appendix A.

## 3. Results

Four sources were included: two retrospective clinical studies, one case report, and one preclinical study. One full-text report was excluded because infliximab was administered intraperitoneally rather than intra-articularly; despite a TMJ-related bite-force endpoint, this route entails systemic exposure and cannot inform the joint-specific exposure, residence, or local safety of direct TMJ delivery [21]. The study-selection flow diagram is provided in the Appendix A.

Although the search encompassed antibodies targeting TNF-α, NGF, and interleukins, all included studies investigated local TNF-α blockade. Human studies evaluated infliximab in juvenile idiopathic arthritis or psoriatic arthritis, whereas the preclinical study used an anti-rabbit TNF-α monoclonal antibody. Study characteristics and principal findings are summarized in Table 1.

In children with juvenile idiopathic arthritis, intra-articular infliximab was well tolerated, but mandibular function and MRI findings showed no consistent improvement [6]. A subsequent study reported progression of acute and chronic MRI changes in most patients [7]. In a patient with psoriatic arthritis, repeated injections were associated with sustained symptomatic improvement without radiographic progression or adverse reactions [18]. In the rabbit model, local TNF-α blockade reduced inflammatory mediators, nociceptive sensitivity, synovitis, and cartilage degeneration [22].

Symptomatic improvement was confined to the single adult case report [18]. In pediatric cohorts, short-term tolerability was not accompanied by consistent structural or functional efficacy; persistent or worsening MRI abnormalities predominated [6,7], although some joints improved [6]. The rabbit model provides mechanistic support for local TNF-α inhibition [22], but the clinical evidence neither establishes efficacy nor excludes treatment-related structural risk.

## 4. Discussion

The currently available evidence on intra-articular monoclonal antibody administration for temporomandibular joint disorders remains limited, but it highlights the potential relevance of immune-cell modulation and cytokine-mediated signaling in temporomandibular joint pathology. The identified studies focused mainly on local TNF-α inhibition in inflammatory temporomandibular joint conditions, particularly juvenile idiopathic arthritis and psoriatic arthritis [6,7,18]. Additional experimental support was provided by a rabbit model of antigen-induced temporomandibular joint arthritis [22]. Symptomatic improvement was confined to one adult case [18], whereas pediatric cohorts showed persistent or worsening MRI abnormalities in most joints [6,7], providing no evidence of structural efficacy. The uncontrolled designs and refractory populations preclude attributing progression to infliximab. Nevertheless, because infliximab is a full-length chimeric IgG1 antibody formulated for intravenous use and anti-drug antibodies are recognized during anti-TNF therapy in JIA [14], Fc-mediated immune-complex effects and uncharacterized intra-articular retention or clearance remain theoretical safety concerns.

The available clinical evidence also substantially narrows the population that might potentially benefit from this approach. Existing reports are limited to immune-mediated inflammatory TMJ arthritis associated with juvenile idiopathic arthritis or psoriatic arthritis [6,7,18]. Accordingly, the most plausible candidates for future clinical studies would be highly selected patients with objectively active TMJ synovitis, preferably confirmed by contrast-enhanced MRI, and persistent pain, functional impairment, or progressive structural changes despite optimized systemic rheumatologic management and established local treatments. Even in this population, intra-articular monoclonal antibody administration should remain experimental and be evaluated in prospective studies with close imaging and safety surveillance. There is currently no direct evidence supporting this approach for primary degenerative TMJ osteoarthritis, internal derangement, or predominantly myogenous TMD, and potential efficacy in inflammatory arthritis should not be extrapolated to these biologically distinct conditions.

The biological rationale for this approach is supported by evidence concerning the role of inflammatory mediators in temporomandibular joint disorders. Increased levels of inflammatory cytokines have been identified in TMJ synovial fluid and tissues, supporting the importance of local inflammatory signaling in joint pathology [2]. Molecular biomarkers have also been linked to inflammatory activity and pain modulation in temporomandibular disorders [10]. In addition, TNF-α, IL-1β, and IL-6 contribute to synovial inflammation, cartilage degradation, and nociceptive sensitization within the TMJ microenvironment [11]. These findings provide mechanistic support for selective cytokine inhibition as a therapeutic strategy.

Beyond cytokine overexpression, activated T cells and other immune-cell populations may contribute to persistent synovial inflammation and abnormal molecular signaling within the TMJ. Dysregulated T-cell responses may sustain cytokine release, osteoclast activation, and cartilage degradation, particularly in immune-mediated disorders such as juvenile idiopathic arthritis and psoriatic arthritis [2,6,7,11]. This broader immune context may help explain why local cytokine blockade could be relevant in selected inflammatory phenotypes, while also indicating that treatment response is likely to depend on disease mechanism and patient selection.

The potential of biologic modulation is also supported by evidence from other musculoskeletal and inflammatory conditions. Anti-TNF-α treatment has been associated with improvement in TMJ-related manifestations in patients with rheumatoid arthritis [3], while TNF-α inhibitors have reduced inflammatory activity and improved MRI findings in inflammatory joint disease involving the hip [13]. Intra-articular monoclonal antibody administration has also been described as a developing strategy in osteoarthritis, with the potential to achieve localized modulation of inflammatory pathways within the joint environment [12]. These observations support the translational rationale for local administration, although findings from other joints cannot be directly extrapolated to the TMJ.

Translation is also constrained by intra-articular drug delivery. Full-length mAbs such as infliximab are approximately 150 kDa, whereas native synovial fluid volume is minimal and reported capacities of the upper and lower TMJ compartments are only about 1.2 mL and 0.5–0.9 mL, respectively [8,9]. Large molecular size does not ensure sustained exposure: lymphatic egress may limit residence, and TMJ-specific clearance remains unknown. The 0.5–1.0 mL injections used in the included studies therefore document administered dose rather than sustained target exposure [6,7,18]. Hydrogels or biodegradable microparticles could prolong local release, whereas antigen-binding fragments or nanobodies may improve tissue penetration but may also clear faster; these approaches require TMJ-specific dose–volume, distribution, pharmacokinetic, and tolerability studies [12].

Hypothesis-generating targets for future intra-articular research include anti-NGF and anti-CGRP antibodies. Neither anti-NGF nor anti-CGRP antibodies have been evaluated by intra-articular administration in the TMJ; available evidence for these agents is limited to systemic administration and/or non-TMJ conditions. Systemic anti-NGF antibodies improved pain and function in hip and knee osteoarthritis but were associated with joint-safety events, including rapidly progressive osteoarthritis [16,23,24,25,26]. CGRP has a TMJ-specific preclinical rationale, but systemic erenumab did not significantly improve TMD-related myalgia [17,27,28]. These targets should therefore be considered hypothesis-generating and require dedicated intra-articular efficacy, pharmacokinetic, and structural-safety studies.

Despite these observations, the evidence remains insufficient to support routine clinical use of intra-articular monoclonal antibodies in temporomandibular joint disorders. The available studies are characterized by small sample sizes, heterogeneous populations and protocols, retrospective or uncontrolled designs, and inconsistent outcome reporting [6,7,18,22]; additionally, the two Stoll reports originated from the same center and included partially overlapping cohorts of unreported extent, reducing the independence of the evidence base [6,7]. The principal strength of this paper is the integration of clinical and preclinical evidence within a structured and transparent framework. Nevertheless, the limited number of studies, variation in dosing and disease context, incomplete reporting, omission of subscription-based databases such as Embase, Scopus, and Web of Science, and the possibility of missed non-indexed or unpublished evidence substantially restrict the strength of the conclusions. Although restricting the search to freely accessible platforms minimized access-related barriers to independent replication and provided broad complementary coverage, it may have reduced retrieval sensitivity compared with a search incorporating these subscription-based databases.

Compared with the broad, usually temporary effects of corticosteroids and the non-targeted mechanical or multicomponent modulation provided by hyaluronic acid, platelet-rich plasma, and autologous conditioned serum, monoclonal antibodies offer predefined pathway specificity, although their TMJ residence and duration of action remain unknown [6,7,8,9,12,18,19,20,22]. Safety and production also differ: hyaluronic acid and corticosteroids have better-characterized local profiles; autologous products require patient-specific processing and vary in composition; and standardized monoclonal antibodies are complex and costly to manufacture, with undefined TMJ-specific immunogenic and Fc-mediated risks [12,14,15]. Comparative efficacy and cost-effectiveness data are unavailable, so superiority over established injectables cannot currently be inferred.

Before clinical testing, candidate formulations could be evaluated through a staged translational pathway comprising assessments of structural stability and target binding; dose-dependent cytotoxicity, inflammatory activation, cellular uptake, and matrix effects in human TMJ synoviocytes and chondrocytes; and, where feasible, confirmation in three-dimensional synovium–cartilage cocultures or joint-on-a-chip systems. Within these systems, target-matched full-length IgG, Fc-silent variants, and scFv formats could be compared at equivalent molar exposure, measuring FcγR engagement, immune-complex formation, complement activation, cytokine release, macrophage activation, and tissue injury to test whether the Fc domain contributes to secondary local inflammation. Subsequent dose-ranging studies might use rabbit arthritis and/or porcine TMJ models. Validated fluorescently or radiolabeled mAbs could be compared across dose levels after single and repeated injections, with relevant endpoints including synovial concentration–time profiles and local half-life, plasma exposure, tissue penetration, biodistribution, target engagement, immunogenicity, and histological safety. Candidates showing acceptable stability, exposure, and safety could then be considered for a small randomized pilot—for example, approximately 20 patients—with MRI-confirmed refractory inflammatory TMJ arthritis, a standard-of-care comparator, blinded clinical and MRI assessment, and follow-up of approximately 12 months.

## 5. Conclusions

Intra-articular monoclonal antibody therapy is biologically plausible for selected inflammatory TMJ disorders, but the supporting evidence remains highly limited and heterogeneous. Preliminary findings suggest possible anti-inflammatory and symptomatic effects, particularly from TNF-α inhibition, without demonstrating consistent clinical or structural benefit. Further clinical testing should be considered only after TMJ-specific formulation stability, human-cell and three-dimensional model testing, pharmacokinetic characterization, and repeated-dose safety studies meet predefined translational criteria, and should then be restricted to refractory immune-mediated TMJ arthritis rather than degenerative, internal-derangement, or myogenous TMD phenotypes. Accordingly, intra-articular monoclonal antibody therapy remains experimental, and the current evidence should be regarded as hypothesis-generating rather than practice-changing.

## Figures and Tables

**Table 1:** Characteristics of the included studies.

Author, Year	Design and Population/Model	TMJ Context and Agent	Key Features of Administration	Follow-Up	Outcomes	Main Findings
Stoll, M.L. et al., 2015 [7]	Retrospective study; children with JIA	Refractory TMJ arthritis; infliximab	Bilateral injection; typically 5–10 mg per TMJ (0.5–1.0 mL); individual volumes not recorded	Median 9 months (range 2–27; first-to-last MRI)	Quantitative MRI assessment of acute and chronic changes	Median acute and chronic MRI scores worsened by 0.25 and 0.75, respectively, indicating progression in most children
Stoll, M.L. et al., 2013 [6]	Retrospective study; 24 children with JIA	Chronic progressive TMJ arthritis refractory to corticosteroid injections; infliximab	Bilateral injection after saline lavage; 0.5–1.0 mL at 10 mg/mL	Mean 7.8 months (SEM 0.6; after initial injection)	Safety, maximal interincisal opening, and MRI findings	No adverse events were reported and maximal interincisal opening was unchanged; MRI abnormalities persisted or progressed, although arthritis resolved in six TMJs
Ohtani, T. et al., 2012 [22]	Preclinical study; 41 rabbits with antigen-induced bilateral TMJ arthritis	Experimental TMJ arthritis; anti-rabbit TNF-α monoclonal antibody	0.2 mL at 10 μg/mL into one TMJ; saline or IgG comparator	21 days (assessments on Days 1, 3, 7, and 21)	Inflammatory mediators, nociceptive response, histopathology, and systemic exposure	Local TNF-α blockade reduced synovial TNF-α, transiently reduced IL-1β, increased withdrawal thresholds, and limited inflammatory and cartilage changes; systemic levels were undetectable
Alstergren, P. et al., 2008 [18]	Case report; 62-year-old woman with psoriatic arthritis	Severe bilateral TMJ symptoms refractory to systemic infliximab and intra-articular glucocorticoids; infliximab	Bilateral 0.5 mL injections at 10 mg/mL every 6 weeks for 36 weeks	36 weeks	Pain, mandibular function, radiographic progression, and safety	Symptoms and mouth opening improved, with sustained benefit over 36 weeks; no radiographic progression or adverse reactions were reported

Abbreviations: IgG, immunoglobulin G; IL-1β, interleukin-1 beta; JIA, juvenile idiopathic arthritis; MRI, magnetic resonance imaging; SEM, standard error of the mean; TMJ, temporomandibular joint; TNF-α, tumor necrosis factor alpha.

**Figure A1:**
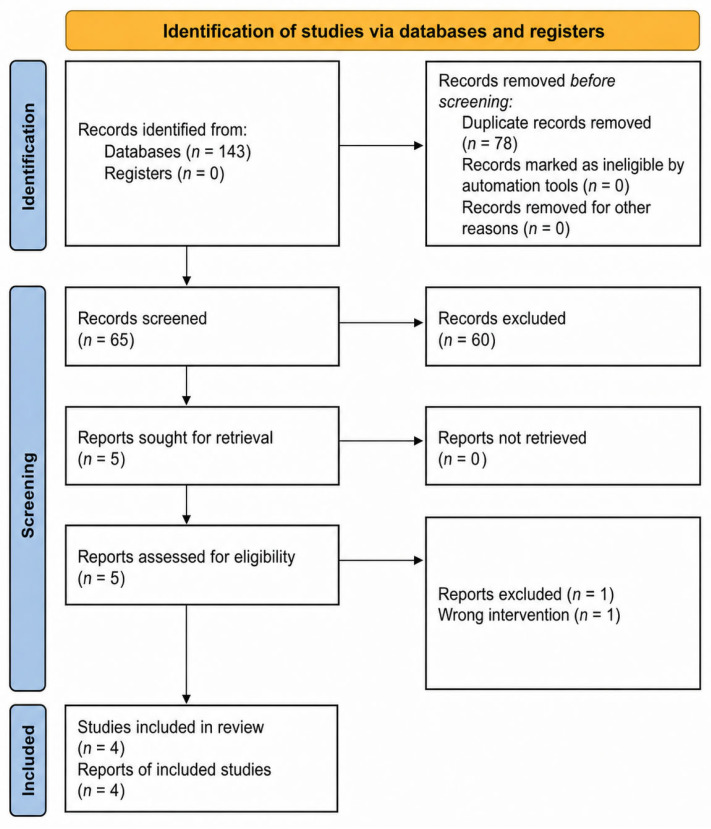
Flow diagram of source identification, screening, eligibility assessment, and inclusion.

## Data Availability

All data underlying this review are available in the cited publications. Extracted data and methodological details are presented in the article and the Appendix A.

## Abbreviations

BASE, Bielefeld Academic Search Engine; CGRP, calcitonin gene-related peptide; IgG, immunoglobulin G; IL, interleukin; JBI, Joanna Briggs Institute; JIA, juvenile idiopathic arthritis; mAb, monoclonal antibody; MRI, magnetic resonance imaging; NGF, nerve growth factor; OSF, Open Science Framework; PRISMA-ScR, Preferred Reporting Items for Systematic Reviews and Meta-Analyses Extension for Scoping Reviews; SYRCLE, Systematic Review Centre for Laboratory Animal Experimentation; TMD, temporomandibular disorders; TMJ, temporomandibular joint; TNF-α, tumor necrosis factor alpha.

## Appendix A. Detailed Methods

### Appendix A.1. Protocol and Reporting

The protocol was prospectively registered in the Open Science Framework (OSF; osf.io/tdfra). The evidence synthesis was conducted and reported in accordance with the PRISMA Extension for Scoping Reviews (PRISMA-ScR) [29].

### Appendix A.2. Eligibility Criteria

Eligible sources included randomized and non-randomized clinical trials, observational studies, case reports, and preclinical animal or in vitro studies evaluating intra-articular administration of monoclonal antibodies into the temporomandibular joint (TMJ). Eligible sources evaluated the actual intra-articular administration of monoclonal antibodies into the TMJ. Studies of systemic monoclonal antibody therapy without intra-articular administration were excluded, even when TMJ-related outcomes were reported.

Eligible human populations comprised patients of any age with inflammatory or degenerative TMJ disorders or TMD-related pain. Relevant experimental models of TMJ inflammation or degeneration were also considered. Studies addressing other joints without TMJ-specific data were excluded.

Eligible interventions included intra-articular monoclonal antibodies targeting TNF-α, nerve growth factor, interleukins, or other inflammatory or nociceptive pathways. Combined interventions were eligible only when the effects attributable to the monoclonal antibody could be distinguished. Any comparator was accepted, including placebo, corticosteroids, hyaluronic acid, systemic therapy, or no comparator.

Eligible outcomes included pain, mandibular function, maximal interincisal opening, other patient-reported outcomes, imaging findings, inflammatory biomarkers, histopathological changes, nociceptive measures, and local or systemic adverse events. No restrictions were applied to publication date, language, clinical setting, geographic location, or follow-up duration.

Reviews, editorials, other non-primary publications, and conference abstracts without sufficient outcome data were excluded. Full texts that could not be obtained, translated, or adequately assessed were also excluded.

### Appendix A.3. Information Sources and Search Strategy

PubMed (MEDLINE), Europe PMC, and BASE were searched from inception to 7 April 2026. Database selection was deliberately limited to freely accessible search platforms to facilitate independent replication without reliance on institutional subscriptions. PubMed provided established biomedical indexing, Europe PMC extended retrieval to full-text-indexed and additional life-science content, and BASE provided broad multidisciplinary coverage as a metadata aggregator indexing more than 400 million records from over 11,000 sources [30]. Reference lists of included studies and relevant reviews were screened for additional eligible sources. Corresponding authors were contacted when full texts or clarifying information were required.

The search syntax was adapted to the requirements of each database. The core search strategy was as follows:

(temporomandibular OR “temporomandibular joint” OR TMJ OR TMD) AND (“monoclonal antibody” OR mAb OR mAbs OR infliximab OR adalimumab OR certolizumab OR golimumab OR tanezumab OR “anti-NGF” OR “anti-TNF” OR “anti-IL” OR “interleukin inhibitor”) AND (“intra-articular” OR intraarticular OR “intra articular” OR intra-articularly OR injection OR injections OR infiltrat*).

### Appendix A.4. Source Selection

All identified records were imported into Rayyan, where duplicates were removed. Before formal screening, the reviewers completed a calibration exercise to standardize the application of the eligibility criteria.

Titles and abstracts were independently and blindly screened by two reviewers (J.K. and O.J.). The same reviewers subsequently assessed potentially eligible full texts. Disagreements were resolved through discussion and consensus. Reasons for exclusion at the full-text stage were recorded. The selection process is presented in Figure A1.

### Appendix A.5. Data Charting

Data were independently charted by two reviewers (J.K. and O.J.) using a standardized and pilot-tested form. Discrepancies were resolved by consensus.

The charted variables included study design, population or experimental model, TMJ disease context, investigated monoclonal antibody, biological rationale, administration characteristics, outcomes, follow-up, safety findings, and principal results.

### Appendix A.6. Critical Appraisal

Critical appraisal was conducted to support interpretation of the limited and methodologically heterogeneous evidence base. The SYRCLE Risk of Bias tool and the relevant JBI critical appraisal checklists for case reports and case series were applied [31,32,33].

Detailed appraisal results are presented in Table A1, Table A2 and Table A3.

**Table A1:** SYRCLE risk-of-bias assessment of Ohtani et al., 2012 [22].

Question	Assessment
1. Sequence generation	Low risk
2. Baseline characteristics	Low risk
3. Allocation concealment	Unclear risk
4. Random housing	Unclear risk
5. Blinding (caregivers/investigators)	High risk
6. Random outcome assessment	Low risk
7. Blinding (outcome assessors)	Low risk
8. Incomplete outcome data	Low risk
9. Selective outcome reporting	Low risk
10. Other sources of bias	Low risk

**Table A2:** Critical appraisal according to the JBI checklist for Case Series.

Question	Stoll, M.L. et al., 2013 [6]	Stoll, M.L. et al., 2015 [7]
1. Were there clear criteria for inclusion in the case series?	Yes	Yes
2. Was the condition measured in a standard, reliable way for all participants?	Unclear	Yes
3. Were valid methods used for identification of the condition?	Yes	Yes
4. Did the case series have consecutive inclusion of participants?	Unclear	Unclear
5. Did the case series have complete inclusion of participants?	Yes	Unclear
6. Was there clear reporting of demographics?	Yes	Yes
7. Was there clear reporting of clinical information?	Yes	Yes
8. Were outcomes clearly reported?	Yes	Yes
9. Was there clear reporting of presenting site(s)/clinic(s)?	Yes	Yes
10. Was statistical analysis appropriate?	Unclear	Yes

**Table A3:** Critical appraisal according to the JBI checklist for Case Reports in Alstergren, P. et al., 2008 [18].

Question	Assessment
1. Were patient’s demographic characteristics clearly described?	Yes
2. Was the patient’s history clearly described and presented as a timeline?	Yes
3. Was the current clinical condition of the patient on presentation clearly described?	Yes
4. Were diagnostic tests or assessment methods and the results clearly described?	Yes
5. Was the intervention(s) or treatment procedure(s) clearly described?	Yes
6. Was the post-intervention clinical condition clearly described?	Yes
7. Were adverse events (harms) or unanticipated events identified and described?	Yes
8. Does the case report provide takeaway lessons?	Yes

### Appendix A.7. Synthesis

Because of the small number and substantial heterogeneity of the included sources, findings were synthesized descriptively. Clinical and preclinical evidence were considered separately. The synthesis focused on the biological rationale for local monoclonal antibody administration, administration characteristics, clinical and imaging outcomes, inflammatory and histopathological findings, nociceptive effects, and safety. Quantitative synthesis was not performed because of differences in study design, populations, interventions, comparators, and outcome reporting.
